# The efficacy of platelet activation indicators for the diagnosis of tubal ectopic pregnancy

**DOI:** 10.12669/pjms.313.7362

**Published:** 2015

**Authors:** Fatma Eskicioglu, Guluzar Arzu Turan, Esra Bahar Gur

**Affiliations:** 1 Fatma Eskicioglu, Department of Obstetrics and Gynecology, Merkez Efendi Hospital, 45050 Manisa, Turkey; 2 Guluzar Arzu Turan, Department of Obstetrics and Gynecology, Şifa University, School of Medicine, 35100 Izmir, Turkey; 3 Esra Bahar Gur, Department of Obstetrics and Gynecology, Şifa University, School of Medicine, 35100 Izmir, Turkey

We read with great interest the article “Can mean platelet volume and platelet distrubition width be possible markers for ectopic pregnancy and tubal rupture?” by Ulkumen et al. in Pakistan Journal of Medical Sciences.^1^ We appreciate the authors for their study. We wanted to contribute to this study as it was related to our article: “The efficacy of complete blood count parameters in the diagnosis of tubal ectopic pregnancy” in Ginekologia Polska.^2^

Ulkumen et al.^1^, aimed to investigate the relationship between platelet activation indices and ectopic pregnancy (EP). They cannot find statistically significant difference in either rupture or non-rupture EP in terms of platelet (PLT), mean PLT volume (MPV) ve PLT distribution width (PDW) levels. However, they claimed that MPV levels was lower, while PDW levels was higher in EP. They showed the high grade inflammation at the implantation site of EP as a cause for both increased PDW level and decreased MPV levels. MPV is a simple PLT activation index. PDW is a stronger sign of platelet activation. The combined use of MPV and PDW could predict activation of PLT more efficiently.^3^ We showed no statistically significant difference between EP (8.38±0.97) and intrauterine pregnancy (8.69±1.14) in terms of MPV levels in our study. We found out that PDW was lower in EP (11.55±1.78) compared to intrauterine pregnancy (16.36±3.00) (*p*<0.001). Soluble factors released from active PLTs increase the invasion capacity of the trophoblast. In this way, PLTs enable maternal spiral arteries to transform into low-resistance large-caliber veins.^4^ In intrauterine pregnancy, its trophoblastic growth and differentiation are realized by cytokines which are released from the trophoblasts, endometrium and decidual stromal cells. However, lack of decidualization is a characteristic feature of EP, unlike in intrauterine pregnancies. Thus, EP invasion may be rather limited when compared to intrauterine pregnancies. EP needs less PLT activation. While, endometrial invasion in the intrauterine pregnancy needs more PLT activation,^2^ as a consequence, PDW values may decrease in EP which necessitates less PLT activation when compared to intrauterine pregnancy.
